# Judaism as a Group Evolutionary Strategy: A Critical Analysis of Kevin MacDonald’s Theory

**DOI:** 10.1007/s12110-018-9310-x

**Published:** 2018-03-10

**Authors:** Nathan Cofnas

**Affiliations:** Balliol College, Oxford, OX1 3BJ UK

**Keywords:** Jews, Anti-Semitism, Group conflict, Gene-culture coevolution, Kevin MacDonald, *Culture of critique*

## Abstract

MacDonald argues that a suite of genetic and cultural adaptations among Jews constitutes a “group evolutionary strategy.” Their supposed genetic adaptations include, most notably, high intelligence, conscientiousness, and ethnocentrism. According to this thesis, several major intellectual and political movements, such as Boasian anthropology, Freudian psychoanalysis, and multiculturalism, were consciously or unconsciously designed by Jews to (a) promote collectivism and group continuity among themselves in Israel and the diaspora and (b) undermine the cohesion of gentile populations, thus increasing the competitive advantage of Jews and weakening organized gentile resistance (i.e., anti-Semitism). By developing and promoting these movements, Jews supposedly played a *necessary* role in the ascendancy of liberalism and multiculturalism in the West. While not achieving widespread acceptance among evolutionary scientists, this theory has been enormously influential in the burgeoning political movement known as the “alt-right.” Examination of MacDonald’s argument suggests that he relies on systematically misrepresented sources and cherry-picked facts. It is argued here that the evidence favors what is termed the “default hypothesis”: Because of their above-average intelligence and concentration in influential urban areas, Jews in recent history have been overrepresented in all major intellectual and political movements, including conservative movements, that were not overtly anti-Semitic.

In the 1990s, Kevin MacDonald wrote a trilogy of books arguing that Judaism is a “group evolutionary strategy,” and the pursuit of this strategy by Jews had far-reaching consequences for world history. In *A People That Shall Dwell Alone: Judaism as a Group Evolutionary Strategy* (1994) he proposed that, since its inception, Judaism has promoted eugenic practices favoring high intelligence, conscientiousness, and ethnocentrism. As a consequence, the contemporary Jewish population (at least the Ashkenazi population) is marked by a high level of these traits, including a mean IQ of 117 (weighted on verbal intelligence). In *Separation and Its Discontents: Toward an Evolutionary Theory of Anti-Semitism* (1998b) he argued that anti-Semitism is a reaction by gentiles to competition for resources with less populous but more organized and competent Jewish groups. In *The Culture of Critique: An Evolutionary Analysis of Jewish Involvement in Twentieth-Century Intellectual and Political Movements* (1998a), he argued that post-Enlightenment Jews who abandoned the religion of Judaism invented a substitute: liberal political, intellectual, and scientific movements with the same social and organizational structure as Judaism, and the same ultimate purpose to promote the evolutionary success of Jews.

According to *The Culture of Critique*, the most influential of these intellectual movements—Boasian anthropology, Freudian psychoanalysis, and Frankfurt School critical theory—were headed by charismatic and authoritarian leaders (analogous to rabbis), they placed great value on verbal brilliance and internal consistency rather than testability or agreement with external reality (analogous to Talmudic scholarship), and they promoted Jewish group interests at the expense of gentiles. The movements advocated separatism and ethnocentrism for Jews, discouraged ethnic identification among white gentiles (in order to prevent group consciousness among white gentiles that might lead to a sense of competition with Jews and thus anti-Semitism), undermined and destabilized traditional European culture to weaken resistance to Jewish control, “pathologized” anti-Semitism, and denied that Jewish behavior plays a role in anti-Jewish attitudes.

MacDonald argues that Jewish intellectual and political movements were responsible for major trends in twentieth-century scientific, political, and demographic history. These movements, he says, were responsible for the rejection of Darwinian thinking among most mainstream social scientists, and also for large-scale nonwhite immigration to European and European-colonized countries (the United States, Australia, etc.).

## Do MacDonald’s Theories Merit Scholarly Attention?

MacDonald’s books received some positive and some mixed reviews. Eysenck (1995) called *A People That Shall Dwell Alone* “a potentially very important contribution.” Masters (1996), while enthusiastic about the prospect of analyzing religions as evolutionary strategies, raised concerns about the depth of the author’s familiarity with the history of religion. Figueredo (1999) gave *Separatism and Its Discontents* a generally positive assessment. In a favorable review of *The Culture of Critique*, Salter (2000) attributed “much of the criticism of MacDonald [to] ignorance of his scholarship and a confounding of political and scientific issues.”

MacDonald’s work on Judaism did not receive widespread attention until the year 2000 when *Slate* journalist Shulevitz (2000) used it in an effort to discredit evolutionary psychology. In response to Shulevitz’s challenge that scientists “can’t ignore bad ideas,” Pinker pointed out that they have no choice. It is impossible to do battle against all bad ideas, of which there are a thousand for every good one.

[D]oing battle against some of them is a tacit acknowledgement that those have enough merit to exceed the onerous threshold of attention-worthiness. MacDonald’s ideas, as presented in summaries that would serve as a basis for further examination, do not pass that threshold . . . (Pinker 2000: unpaginated).

Given this fact—that ideas need to meet an “onerous threshold of attention-worthiness”—what justifies giving attention to MacDonald’s theories, published two decades ago and, with just a few exceptions (e.g., favorable treatment in Wilson 2002; criticisms in Atran 2002:230–33), largely ignored in mainstream literature?

Even if Pinker was right that MacDonald’s theories did not have enough prima facie merit to warrant attention in 2000, developments in the past 18 years have changed the situation. There are at least three reasons to give MacDonald a hearing.

First, some respected psychologists and evolutionary theorists have reported that they found value in MacDonald’s work. For example, David Sloan Wilson endorsed the ideas in *A People That Shall Dwell Alone* and strongly criticized the representatives of the Human Behavior and Evolution Society who rejected MacDonald: “Even evolutionary psychologists, who have experienced their share of persecution in academic circles, seem more concerned to protect their own reputations than to defend the work of their colleague” (quoted in Salter 2000). Wilson is also thanked in the acknowledgments sections of all three books. And, as noted, Eysenck, Figueredo, Salter, and others have publicly given positive evaluations of some or all of MacDonald’s trilogy. There are also a number of serious scholars who are attracted to MacDonald’s ideas but will not endorse or even comment on them publicly because they fear that they will be perceived as anti-Semitic. This amounts to at least some degree of prima facie evidence that MacDonald’s theory should be considered.

Second, it is an undeniable fact that, in the past few hundred years, Jews have had a disproportionate influence on politics and culture in the Western world, if not the whole world. It might be worthwhile to investigate this phenomenon from a biosocial or evolutionary perspective. So far there have been only a handful of such investigations, including Cochran et al.’s (2005) study on the evolution of Jewish intelligence, Dunkel et al.’s (2015) report of high mean levels of the “general factor of personality” among Jews, and, what is by far the most ambitious, MacDonald’s (1994, 1998a, 1998b). The idea that Jewish influence resulted, at least in some cases, from their pursuit of a group evolutionary strategy cannot be dismissed a priori. Since MacDonald has defended this theory, he seems to provide a starting point for anyone wishing to investigate the understudied issue of Jewish influence. If he is wrong, it may be useful to know why and how.

Third and perhaps most important, though, is that MacDonald’s work *has* been influential—enormously so—in a certain segment of the lay community, namely, among anti-Semites and adherents of the burgeoning movement known as the “alt-right.” It is hard to overstate his influence among this group. Some years ago Derbyshire (2003) called him “the Marx of the anti-Semites,” and with the advent of the alt-right his audience has grown substantially. Richard Spencer, whom the *New York Times* calls “the leading ideologue of the alt-right movement” (Goldstein 2016), introduced MacDonald at a conference with one sentence: “There is no man on the planet who has done more for the understanding of the pole around which the world revolves than Kevin MacDonald” (Spencer 2016). Andrew Anglin, who runs the most popular alt-right/neo-Nazi website, says in his “Guide to the Alt-Right” that “MacDonald’s work examining the racial nature of Jews is considered crucial to understanding what the Alt-Right is about” (Anglin 2016). The *New York Times* describes MacDonald’s trilogy as “a touchstone” for the alt-right, a movement encompassing hundreds of thousands, if not millions, of people (Caldwell 2016). MacDonald is also editor of the *Occidental Observer*, a fairly popular magazine that is devoted largely to interpreting current events in the light of his theories about Jews. Anglin (2016) lists the *Occidental Observer* as one of eight “sites and people” playing a key role in the alt-right movement.

The refusal of scholars to engage with MacDonald has had unintended negative consequences. Many of his enthusiasts see him as credible because there has never been a serious academic refutation of his theories. The strategy employed 18 years ago—declaring his work to be anti-Semitic and/or to not reach the threshold to warrant scholarly attention—had the doubly unfortunate effect of intimidating scholars with a legitimate interest in the topic of Jewish evolution and behavior, and creating a perception among some laypeople—even if it was false—that MacDonald was being persecuted by the academic community.

This paper attempts to give MacDonald’s theories a fair hearing. It focuses on the argument of *The Culture of Critique*. This book builds on the previous two, so the whole trilogy stands or falls on its merits. It has also been the most influential of the three books. It defends a radical, interesting hypothesis, and the topic it addresses (Jewish overrepresentation in intellectual movements) is worthy of study in any case. The conclusion, however, will be that the argument of *The Culture of Critique* is built on misrepresented sources and cherry-picked facts. The evidence actually favors a simpler explanation of Jewish overrepresentation in intellectual movements involving Jewish high intelligence and geographic distribution.

## Jewish High IQ and Geography: An Alternative, Simpler Theory That Explains More of the Data?

The mean Ashkenazi Jewish IQ appears to be around 110 (Lynn and Kanazawa 2008)—moderately lower than MacDonald’s estimate of 117. Jewish intellectual accomplishment is consistent with higher mean intelligence. The basic facts are well known. For example, though never more than 3% of the US population (Pinker 2006), Jews constitute 31% of US Nobel laureates in chemistry, 50% in economics, 37% in physics, 39% in physiology or medicine, and 33% in literature (jinfo.org). Lynn and Kanazawa (2008) give a good review of their overrepresentation in high-IQ occupations and in leadership positions in the arts, science, and industry.

But a mean IQ of 110 is not enough to explain Jewish achievement (Nisbett 2009:181). It is likely that Jews also have a geographic advantage. Since the Enlightenment and particularly in the twentieth century, European Jews have been highly concentrated in major urban centers (Warsaw, Berlin, Frankfurt, Vienna, Paris, New York City, Los Angeles, etc.). These areas tended to have the infrastructure to support intellectual achievement. Indeed, even without postulating high Jewish IQ, from their location alone we would expect them to be overrepresented in intellectual endeavors.

The combination of the aforementioned factors suggests an alternative theory—what could be called the “default hypothesis”—of Jewish involvement in twentieth-century liberal movements, namely: Because of Jewish intelligence and geography—particularly intelligence—Jews are likely to be overrepresented in any intellectual movement or activity that is not overtly anti-Semitic. The qualification that Jews are not overrepresented in *overtly anti-Semitic* movements is important because, in the twentieth century, a higher proportion of right-wing than left-wing movements were overtly anti-Semitic. According to the default hypothesis, Jewish involvement in politics has been somewhat skewed to the left in recent history, but Jews are also overrepresented in right-wing movements that are not anti-Semitic.

The default hypothesis seems to have more explanatory power and to be more parsimonious than MacDonald’s because it posits only two factors—IQ and geography—to explain Jewish overrepresentation in *all* (non-overtly anti-Semitic) intellectual activities: The two factors explain why Jews are more than half of world chess champions (Cochran et al. 2005) and why they comprised “[a]lmost one-half” of the elite American intellectuals in Kadushin’s (1974:23) sample (MacDonald 1998a:3). Of course, explanatory power and parsimony are not the only consideration: Agreement with empirical evidence is the ultimate arbiter. This paper attempts to determine whether the evidence favors MacDonald’s thesis or the default one.

The default hypothesis is not tied to any particular explanation of the *cause* of above-average Jewish IQ. Some researchers favor a genetic explanation. In an influential paper, Cochran et al. (2005) argued that during the Middle Ages Ashkenazim were selected for the intellectual ability to succeed in white-collar occupations. However, it is theoretically possible that the Jewish–gentile IQ gap is due at least in part to some yet-to-be-identified cultural factor (Nisbett 2009). Whatever the cause, high Jewish IQ presumably plays a role in Jewish overrepresentation in cognitively demanding activities.

## Overview of Some Problems with the Arguments in *The Culture of Critique*

To review, the claim of *The Culture of Critique* is that “Jewish-dominated intellectual movements were a critical factor (necessary condition) for the triumph of the intellectual left in late twentieth-century Western societies” (MacDonald 1998a:17; see also 214–15).

[I]ndividuals who strongly identified as Jews have been the main motivating force behind several highly influential intellectual movements that have simultaneously subjected gentile culture to radical criticism and allowed for the continuity of Jewish identification. Together these movements comprise the intellectual and political left in this century, and they are the direct intellectual ancestors of current leftist intellectual and political movements, particularly postmodernism and multiculturalism (1988a:213).

While Jewish intellectual movements vary in their details, they have (according to *The Culture of Critique*) the same broad agenda to (a) subject gentile society to radical critique that undermines its traditional institutions, (b) attack white gentile ethnocentrism and unity (in order to weaken the potential for organized gentile resistance to Jewish domination), (c) “pathologize” anti-Semitism and obscure the fact that anti-Jewish attitudes may be a response to Jewish behavior, and (d) promote multiculturalism for white gentiles (in order to weaken gentile power) while promoting separatism for Jews and racial purity in Israel. Jewish movements are alleged to have been responsible for banishing Darwinian thinking from social science, promoting environmentalism as an explanation for individual and group differences in behavior, and proscribing the study of group differences in psychology. As noted, the way in which Jewish intellectual movements are organized—headed by charismatic, authoritarian leaders such as Freud and Boas—is supposedly analogous to the organization of traditional Judaism.

There are several categories of questionable argumentation in *The Culture of Critique*. The following is a preview—more detailed examples will be given in later sections.

### The Same Behavior Is Interpreted Differently When Exhibited by Jews or Gentiles

A common pattern throughout *The Culture of Critique* is that the same behavior is given a different interpretation depending on whether it is performed by Jews or gentiles. For example, when gentiles assume leadership positions in radical movements (e.g., John Dewey, Carl Jung), it is because “gentiles have . . . been actively recruited to the movements . . . and given highly visible roles . . . in order to lessen the appearance that the movements are indeed Jewish-dominated or aimed only at narrow Jewish sectarian interests” (1988a:4). MacDonald calls this phenomenon “a major theme” of his book. Another explanation he gives for gentile involvement in radical politics is that “once Jews have attained intellectual predominance, it is not surprising that gentiles would be attracted to Jewish intellectuals as members of a socially dominant and prestigious group and as dispensers of valued resources” (1988a:3).

Of course, it is *possible* that in all these cases where Jews and gentiles were both involved in radical politics, the Jews were acting as ethnic activists while the gentiles were being manipulated. But this theory requires strong positive evidence to be credible. As shall be argued, MacDonald never provides such evidence.

### Sources Are Cherry-Picked and Jewish Involvement in Anti-Jewish Activism Is Ignored

MacDonald says that “there is a broad Jewish consensus [in the US] on such issues as Israel” (1988a:305). Nowhere in the book does he acknowledge that a great deal of Jewish involvement in politics across time and place has been decidedly opposed to narrow Jewish interests, including Israel. The most influential Jewish radical in history, Karl Marx, held extremely anti-Jewish views (Marx 2010). The most influential Jewish radical alive—when *The Culture of Critique* was published and still to this day—is Noam Chomsky. Chomsky is mentioned one time in *The Culture of Critique*—in an endnote where MacDonald comments simply that he “could . . . be regarded as someone whose writings were not highly influenced by his Jewish identity and specifically Jewish interests” (1988a:154, n. 15). There is no mention of Chomsky’s extreme anti-Israel positions and opposition to Jewish nationalism. George Soros—possibly the most politically influential Jewish financier in the world and a major promotor of liberalism/multiculturalism—dissociates himself from the Jewish community and opposes Jewish interests (as MacDonald conceives them). He is not mentioned once in the book. MacDonald paints a picture of Jews as hypocrites who impose liberalism on gentiles and adopt nationalism for themselves, but he ignores the fact that many of the most influential Jews seem to promote liberalism and multiculturalism for both gentiles and Jews.

Just as problematically, in a number of cases MacDonald fails to report that Jews whom he identifies as ethnic activists took stands against Israel and other Jewish interests (again, defining “Jewish interests” in MacDonald’s terms as ethnic self-preservation).

### The Failure of Jews to Support Overtly Anti-Semitic Movements Is Interpreted as Evidence of Extreme Jewish Ethnocentrism

Many twentieth-century Jews ostensibly abandoned their Jewish identity and sought to assimilate. MacDonald points out that these Jews often did not support gentile nationalist movements—which he acknowledges were anti-Semitic—and he argues that this is evidence that these Jews were insincere in their desire to assimilate and were actually engaging in “Jewish crypsis” (his term).

It is highly questionable whether this inference is justified. Twentieth-century anti-Semitic nationalist movements were generally not welcoming of ethnic Jews regardless of their desire to assimilate. And even if they were, it seems unreasonable to question a Jew’s desire to reject Judaism because he did not want to kill, expel, or oppress his (probably still-Jewish) family and former friends.

### Sources Are Misrepresented

In numerous places in *The Culture of Critique*, references are given to support a claim but no support can be found in the original source, or the original source is misrepresented. Because the present paper is focused on the *argument* of the book, it only reports some of these misrepresentations where they significantly affect the argument. Also, for considerations of length, it only reports cases of mishandling of sources where the problems can be clearly exposed in a reasonable amount of space. Despite the fact that only some instances of mishandling of sources are reported here, these cases alone raise serious questions about MacDonald’s research practices.

### No Evidence Is Ever Acknowledged to Count against the Theory

In many places, MacDonald himself brings up facts that seem to go against the predictions of his theory. While these individual facts may not in themselves necessarily refute his hypothesis, rather than revising his ideas or acknowledging that he cannot explain everything, he dogmatically insists that the apparent counterexamples actually *support* his views.

For example, he claims several times that Jews are opposed to affirmative action because it is against their ethnic interests (1988a:101, 105, n. 16, 308, 313, 315; see also 240–41). He says that affirmative action policies “would clearly preclude free competition between Jews and gentiles” (1988a:101) and, elsewhere, that they “would necessarily discriminate against Jews” (1988a:315). In a parenthetical, he notes that when an anti-affirmative action measure was put on the ballot in California, Jews voted for it “in markedly lower percentages” than other white groups (1988a:311). That is, Jews voted to *support* affirmative action. His explanation for this is that “because of their competitive advantage” among whites, “Jews may perceive themselves as benefiting from policies designed to dilute the power of the European-derived group as a whole on the assumption that they would not suffer any appreciable effect.” Again, he shows a facile tendency to spin an apparent disconfirmation of his theory as actually a verification of it.

### Hundreds of Years of Gentile Radicalism Are Ignored

The reader of *The Culture of Critique* who has no knowledge of history is led to believe that European society was traditionally marked by “hierarchic harmony” (1988a:315) and naive, happy acceptance of traditional religion, institutions, and family relations. Then, after the Enlightenment, Jews emerged from the ghettos and commenced what was to be a 300-year war on the foundations of European culture. MacDonald ignores a long history of radical and critical gentile thought from the ancient Greek philosophers to Rousseau to the Social Gospel Movement to French existentialism to Bill Ayers to Peggy McIntosh and countless other examples.

## Boasian Anthropology, Environmentalism, and Opposition to the Study of Race Differences

The second chapter of *The Culture of Critique* is titled “The Boasian School of Anthropology and the Decline of Darwinism in the Social Sciences.” MacDonald sees Boas as having been a strongly identified Jew who pursued (and distorted) science with the goal of preventing anti-Semitism. Boas and his followers in the 1920s promoted the idea that

American culture [was] overly homogeneous, hypocritical, and emotionally and esthetically repressive (especially with regard to sexuality). Central to [their] program was creating ethnographies of idyllic cultures that were free of the negatively perceived traits that were attributed to Western culture. Among these Boasians, cultural criticism crystallized as an ideology of “romantic primitivism” in which certain non-Western cultures epitomized the approved characteristics Western societies should emulate (1988a:28–29).

This passage and others throughout the chapter suggest that Boasians were the first to romanticize primitive cultures as “idyllic” and not subject to the ills of Western civilization. In reality, by Boas’s time this had been a major theme among many gentile intellectuals for more than 150 years. Jean-Jacques Rousseau, who popularized the romantic image of “savages” in the eighteenth century, is mentioned once in *The Culture of Critique*—in passing, in an endnote (1988a:211, n. 35). According to Rousseau:

The more one reflects on it, the more one finds that this state [of primitive life] was the least subject to upheavals and the best for man, and that he must have left it only by virtue of some fatal chance happening that, for the common good, ought never have happened. The example of savages, almost all of whom have been found in this state, seems to confirm that the human race had been made to remain in it always (Rousseau 2011:74).

Not all eighteenth-century European intellectuals agreed that civilization was a mistake. But virtually all seemed to accept Rousseau’s characterization of primitive life as idyllic. His ideas, including his critique of Western civilization, played an important role in triggering the French Revolution and (later) in the development of socialism, especially by Marx. All in all, he was quite probably the most influential thinker of the eighteenth century in both the short and the long run (Durant and Durant 1967).

So, contrary to what is suggested in *The Culture of Critique*, the tradition of critiquing Western civilization by comparing it unfavorably to traditional cultures was neither developed nor made popular by Jews. But even if he was not its inventor, could it be that Boas promoted the Rousseauian view of “romantic primitivism” to advance Jewish interests? It is true that many of Boas’s students were Jews (e.g., Alexander Goldenweiser, Melville Herskovits, Robert Lowie, Paul Radin, Edward Sapir, and Leslie Spier)—not particularly surprising given the high concentration of Jews at Columbia University at the time. But the most effective and indefatigable “Boasians” were not Jewish. MacDonald (1988a:26) notes that the students of Boas who “achieved the greatest public renown” were the gentiles Ruth Benedict and Margaret Mead. He expounds: “As in several other prominent historical cases . . ., gentiles became the publicly visible spokespersons for a movement dominated by Jews.” According to MacDonald (1988a:27), Boas “strenuously promoted and cited” Benedict and Mead as part of a ruse to hide the fact that the whole movement was designed to promote Jewish interests.

But MacDonald does not supply any compelling reasons to think that Benedict and Mead were under the control of Boas. Even if we accept that Boas’s commitment to Jewish interests biased his science and made him critique Western society and promote environmentalist, culture-based explanations of human behavior, both Benedict and Mead were strong-willed, charismatic iconoclasts who seemed to be self-directed. Although MacDonald sees them as puppets of Boas, another possibility is that Benedict, Mead, and Boas were leaders of a somewhat misguided scientific movement, with Boas being technically the “teacher” because he happened to be a few years older, and Mead being the most influential. Criticizing Boas’s scientific standards, MacDonald says that he “completely accepted” Mead’s conclusions derived from a few months of fieldwork in Samoa and “uncritically allowed Ruth Benedict to distort his own data on the Kwakiutl” (1988a:28). But, taking MacDonald’s description of the facts at face value, this suggests that Mead and Benedict were, at least in these cases, taking the initiative to distort science for ideological ends. Perhaps it was the Jewish Boas who made them do this. Or perhaps, in the absence of compelling evidence to suggest otherwise, both Jews and gentiles occupied leadership roles in this movement in anthropology.

According to *The Culture of Critique*, Boasian anthropology was only the first Jewish salvo against hereditarianism and the study of race. A major theme in the book is that Jews were responsible for tabooing research on race differences, particularly in intelligence. MacDonald ignores the fact that influential gentiles have been well represented among environmentalists studying race differences in intelligence, and Jews have been clearly overrepresented among prominent hereditarians.

MacDonald (1988a:314) approvingly cites Ryan’s (1994:11) speculations on the psychology of the authors of *The Bell Curve* (Herrnstein and Murray 1994):

Herrnstein essentially wants the world in which clever Jewish kids or their equivalent make their way out of their humble backgrounds and end up running Goldman Sachs or the Harvard physics department, while Murray wants the Midwest in which he grew up—a world in which the local mechanic didn’t care two cents whether he was or wasn’t brighter than the local math teacher.

(Incidentally, Ryan’s article was published in the *New York Review of Books*—a journal that MacDonald repeatedly identifies as being an organ of Jewish interests.) This illustrates a blatant double standard applied to Jews and gentiles by MacDonald. The Jewish Richard Herrnstein, then head of the psychology department at Harvard, was the most prominent academic defender of hereditarianism regarding race differences in intelligence since WWII. Instead of accepting that Herrnstein is an example that does not support his thesis, MacDonald spins the facts by implying that Herrnstein supported the theory of race differences in intelligence because it would promote his ethnic interests. In contrast, the gentile Murray is portrayed as having no such sinister motivations—only a wish, in MacDonald’s words, for “a society with harmony among the social classes and with social controls on extreme individualism among the elite” (1988a:314).

A reasonable list of the most high-profile advocates of hereditarianism might be the following: Hans Eysenck, Arthur Jensen, Richard Lynn, Linda Gottfredson, J. Philippe Rushton, and the aforementioned Herrnstein and Murray. Eysenck had a Jewish mother, making him Jewish by both Jewish law and MacDonald’s standards. Jensen was one-quarter Jewish, so he can be counted as a gentile. That means that two out of seven of the most prominent hereditarians were Jewish, making Jews extremely overrepresented in this group relative to their numbers in the general population.

## Freud and Psychoanalysis

According to *The Culture of Critique*, “There is . . . evidence that Freud conceptualized himself as a leader in a war on gentile culture” (1988a:117). Psychoanalysis was a pseudoscientific movement designed to pathologize anti-Semitism and undermine gentile culture and social cohesion by attacking institutions regulating love and sex. “Psychoanalytic assertions [that sexual repression prevented relationships from being based on love and affection] were never any more than speculations in the service of waging a war on gentile culture” (1988a:126). Jews, led in the US by the “New York Intellectuals,” turned Freudianism into a secular religion, using it to attack the philosophical and institutional foundations of Western culture.

Let’s consider first Freud’s influence via the New York Intellectuals. MacDonald notes that of the top 21 American intellectuals according to peer ratings in the 1970s (Kadushin 1974), 15 were Jewish (and most were New York Intellectuals). Eleven of these 15, he says, were “‘significantly influenced by Freudian theory at some point in their careers,’” and 10 of those 11 held “liberal or radical political beliefs at some period of their career” (MacDonald 1998a:141, quoting/citing Torrey 1992:185). The implication is that these influential Jewish intellectuals promoted Freudianism to undermine gentile culture and advance their ethnic interests. But MacDonald leaves out some crucial information.

The 15 Jews among the top 21 intellectuals were (1) Daniel Bell, (2) Chomsky, (3) Irving Howe, (4) Norman Mailer, (5) Robert Silvers, (6) Susan Sontag, (7) Lionel Trilling, (8) Hannah Arendt, (9) Saul Bellow, (10) Paul Goodman, (11) Richard Hofstadter (Jewish father), (12) Irving Kristol, (13) Herbert Marcuse, (14) Norman Podhoretz, and (15) David Riesman. A closer look shows that only two or three of these cases support MacDonald’s thesis, and several are clear counterexamples. First off, five of these intellectuals are, by MacDonald’s criteria, unambiguously anti-Israel and therefore opposed to Jewish interests. Chomsky was (and still is) arguably the world’s leading critic of Israel. Mailer tended to sympathize with the Palestinians (Theodoracopulos 2015). When Sontag accepted the Jerusalem Prize in 2001, she used the occasion to condemn Israel (Cockburn 2001). Marcuse (who will be discussed in more detail below) advocated the return of Arab refugees to Israel, ending Jewish control of the country (Marcuse 2005:181). Arendt was the student, promoter, and lover (in a romantic sense) of the Nazi philosopher Martin Heidegger. She was best known for her book *Eichmann in Jerusalem* (Arendt 1963), in which she argued that Israeli laws were comparable to the Nazi Nuremberg laws and that holocaust-orchestrator Eichmann had been given a “show trial” and was not a particularly bad person (just that he was prompted to do bad things by circumstances beyond his control—though she faults him for not being brave enough to protest). In 1948, Arendt (along with Einstein, Sidney Hook, and 24 other prominent Jews) signed a letter to the *New York Times* which described the political party of Menachem Begin as “closely akin in its organization, methods, political philosophy and social appeal to the Nazi and Fascist parties” (Shatz 2004:65).

Another intellectual on the list, Saul Bellow, was a conservative who opposed feminism, multiculturalism, and political correctness. Bellow urged Allan Bloom, another Jewish academic at the University of Chicago, to write *The Closing of the American Mind* (Bloom 1987), one of the most influential pro-traditionalist academic books in the past few decades (Ahmed and Grossman 2007).

It is ironic that MacDonald casts Robert Silvers as a part of a nefarious Jewish Freudian movement. In the paragraph immediately following the one in which he introduces this list of 15 Jewish intellectuals, MacDonald writes:

The link between psychoanalysis and the political left, as well as the critical role of Jewish-controlled media in the propagation of psychoanalysis, can be seen in the recent uproar [over] Frederick Crews’s critiques of the culture of psychoanalysis. The original articles were published in the *New York Review of Books* . . . (1988a:141).

Silvers is the longtime editor of the *New York Review of Books*, and one of the 11 whom MacDonald identifies as being influenced by Freud.^1^

Bell, Hofstadter, and Riesman were liberals, though not particularly extreme, not known for promoting Freud, and not seriously involved in Jewish causes. (Bell 1962:16 described his perspective as “anti-ideological, but not conservative,” and he criticized utopian schemes such as Marxism as well as aspects of the prevailing social order.) Trilling may have been a nominal Marxist in the 1930s (D. Sidorsky, personal communication), though he evinced little interest in Jewish causes and his ethnic awareness seemed to be triggered mainly when he faced anti-Semitism. Goodman had no apparent interest in his fellow Jews, though he identified as an anarchist, so by MacDonald’s criteria might be considered an enemy of gentile culture. Howe was a liberal who supported as well as criticized Israel. That leaves the neoconservatives Kristol and Podhoretz. Kristol and Podhoretz became decidedly anti-liberal, though later in their careers they openly and aggressively supported Israel, Jewish interests, and, in Podhoretz’s case, unfettered immigration to the US.

The naive reader of *The Culture of Critique* would think that 11 of 15 top Jewish intellectuals were using Freudianism to attack the traditions of gentile culture while promoting separatism for Jews in the US and in Israel. MacDonald makes this conclusion fairly explicit:

Of these [15 Jewish intellectuals], only Noam Chomsky could possibly be regarded as someone whose writings were not highly influenced by his Jewish identity and specifically Jewish interests. The findings taken together indicate that the American intellectual scene has been significantly dominated by specifically Jewish interests and that psychoanalysis has been an important tool in advancing these interests (1988a:154, n. 15—partially quoted earlier).

But the evidence reviewed above suggests that this is a serious distortion of the facts. Even if it is true that 11/15 of these intellectuals were influenced by Freud “at some point in their careers,” virtually none of them comes close to conforming to MacDonald’s paradigm of a Jewish radical. Only one—Podhoretz—could be accused of hypocritically advocating different immigration policies for the US and Israel, though he was/is not a liberal and Freudianism played no meaningful role in his thinking. On the other hand, we clearly find that several people on the list—*a list cited by MacDonald himself to support his thesis*—are serious counterexamples to the theory of Judaism as a group evolutionary strategy. We find on this list possibly the world’s leading critic of Israel (Chomsky), a liberal who advocates the same immigration policies for the US and Israel (Marcuse), a leading advocate of traditional Western values (Bellow), and several others who, to varying degrees, were opposed or indifferent to Israel and Jewish interests.

MacDonald brings voluminous evidence that Freud strongly identified as a Jew. Based on numerous sources, he argues that Freud was unconditionally committed to promoting Jewish interests, that he “pathologized” anti-Semitism, and that he attacked gentile culture because he saw it as a threat to Jews (1988a:146). MacDonald emphasizes numerous times throughout the book that “scientist-activists” like Freud developed theories to show that “Jewish behavior [is] irrelevant to anti-Semitism” (1988a:17; see also 142, 146). He claims that *Moses and Monotheism* “contains several assertions that anti-Semitism is fundamentally a pathological gentile reaction to Jewish ethical superiority,” citing Freud (1967:114–17) (MacDonald 1998a:120). However, while pages 114–17 of this edition of *Moses and Monotheism* do discuss anti-Semitism, there is nothing about ethics/morality at all, let alone the ethical superiority of Jews or Judaism. (MacDonald did not respond to an email asking what he was referring to.)

Although Freud certainly did have a Jewish identity—if only because he was continually reminded of it by anti-Semites—MacDonald does not tell the full story. Consider the following incident (not described in *The Culture of Critique*). In 1929, Jews attempted to erect a partition screen to separate men and women at the Western Wall. In response, Arabs killed 29 Jews in Hebron, which led to riots in which 120 Jews and 87 Arabs were killed. A representative of the Zionist organization Keren Hayesod asked Freud to sign a petition condemning the Arabs for initiating the violence. Freud refused to sign, explaining that the Jews were partly responsible for inviting violence on themselves: “I concede with sorrow that the unrealistic fanaticism of our people is in part to be blamed for the awakening of Arab distrust” (Freud 2004). This episode undermines MacDonald’s caricature of Freud as a monomaniacal activist dedicated to excusing Jewish behavior and pathologizing anti-Semitism.

## The Frankfurt School and Critical Theory

Chapter 5 of *The Culture of Critique* is titled “The Frankfurt School of Social Research and the Pathologization of Gentile Group Allegiances.” It focuses on the alleged hypocrisy of members of the Frankfurt School in advocating for collectivism among Jews in both Israel and the diaspora, and pathologizing any feelings of group allegiance in white gentiles. MacDonald sees the Frankfurt School as having influenced the field of psychology particularly through the publication of *The Authoritarian Personality* (Adorno et al. 1950), a book published as part of a series called *Studies in Prejudice*. He concludes that “the agenda of the Frankfurt School” was to facilitate “radical individualism . . . among gentiles while retaining a powerful sense of group cohesion among Jews” (1988a:215). “[T]he central agenda of *The Authoritarian Personality* is to pathologize gentile group strategies while nevertheless leaving open the possibility of Judaism as a minority group strategy” (1988a:172). The Frankfurt School influenced the humanities through the development of “critical theory.”

The main problem with MacDonald’s argument is that he interprets criticism of nationalism in gentile groups to indicate approval of *Jewish* nationalism as long as the latter is not explicitly condemned. He never cites positive evidence that representatives of the Frankfurt School approved of Jewish nationalism, and he ignores evidence that they in fact disapproved of it. Leaving aside the question of the scholarly merits of the Frankfurt School or *The Authoritarian Personality*, there is no positive evidence that members of the Frankfurt School were hypocrites who condemned collectivism in gentiles and promoted it for Jews.

In his critique of *The Authoritarian Personality*, MacDonald emphasizes “the double standard in which gentile behavior inferred from high scores on the F-scale or the Ethnocentrism Scales is viewed as an indication of psychopathology, whereas precisely the same behavior is central to Judaism as a group evolutionary strategy” (1988a:168). But nowhere does he present evidence that Adorno et al. approved of this behavior in Jews, which is what would be necessary for them to have a “double standard.” MacDonald just assumes that they approve of this behavior because they were Jewish. Regarding the claim in *The Authoritarian Personality* that anti-Semitism is associated with a strong in-group ideology, MacDonald comments that “the implication is that strong ingroup ideologies should be reserved for Jews and are dangerous in others” (1988a:170). But nowhere is this actually stated in *The Authoritarian Personality*. It seems the “implication” is strong in MacDonald’s mind because of the nefarious motives he attributes to the Jewish authors. This does not count as evidence.

To illustrate how *The Authoritarian Personality* is anti-gentile, MacDonald singles out a chapter by R. Nevitt Sanford (who, incidentally, was a gentile). In MacDonald’s words:

R. Nevitt Sanford . . . finds that affiliation with various Christian religious sects is associated with ethnocentrism, and that individuals who have rebelled against their parents and adapted another religion or no religion are lower on ethnocentrism. These relationships are explained as due to the fact that acceptance of a Christian religion is associated with “conformity, conventionalism, authoritarian submission, determination by external pressures, thinking in ingroup-outgroup terms and the like vs. nonconformity, independence, internalization of values, and so forth” (Adorno et al. 1950:220). Again, individuals identifying strongly with the ideology of a majority group are viewed as suffering from psychopathology, yet Judaism as a viable religion would necessarily be associated with these same psychological processes (MacDonald 1998a:174–75).

MacDonald cites Sanford out of context and totally misrepresents his conclusion. First, when Sanford refers to “conformity, conventionalism, authoritarian submission . . .,” he is *not* characterizing Christian belief. He says that to understand the relation between religion and ethnocentrism, we must consider what psychological factors play a role in the individual’s acceptance or rejection, such as “conformity, conventionalism, authoritarian submission.” He is not talking specifically about Christianity, and he says explicitly that these factors do not play a role in “genuine” Christianity. He clearly distinguishes between nominal Christians who adopt the religion of their parents or of the majority simply because they tend to submit to authority, and those “whose religion would appear to be ‘genuine,’ in the sense that it was arrived at more or less independently of external pressure and takes the form of internalized values” (Adorno et al. 1950:220). Sanford says that the latter—the “genuine” Christians—“tend to score low, often very low, on ethnocentrism.”

Second, Sanford characterizes traditional Christianity in a positive, not a negative, way. He refers to “Christian humanism which works against prejudice” (Adorno et al. 1950:215). He writes that “in America today,” the “traditional Christian values of tolerance, brotherhood, and equality” appear to be “more firmly held by people who do not affiliate with any religious group,” though “genuine” Christians low in ethnocentrism “probably predominate in [certain] Protestant denominations” (Adorno et al. 1950:219–20). Thus Sanford identifies the values promoted by the Frankfurt School with *Christianity*, not Judaism.

MacDonald (1998a:240) approvingly cites Jay’s (1973:32) statement on the Frankfurt School: “What strikes the current observer is the intensity with which many of the Institute’s members denied, and in some cases still deny, any meaning at all to their Jewish identities.” MacDonald sees this denial as “crypsis”—members of the Frankfurt School “conceal[ed] their Jewish identities . . . [and] engage[d] in massive self-deception.” Jewish intellectual movements “typically [occur] in an atmosphere of Jewish crypsis or semi-crypsis in the sense that the Jewish political agenda [is] not an aspect of the theory and the theories themselves [have] no overt Jewish content” (1988a:241). But again, in the case of the Frankfurt School, there is no *positive* evidence for this, and MacDonald ignores the evidence against it. For example, in his discussion of Erich Fromm, a leading proponent of the Frankfurt School, MacDonald writes: “The irony (hypocrisy?) is that Fromm and the other members of the Frankfurt School, as individuals who strongly identified with a highly collectivist group (Judaism), advocated radical individualism for the society as a whole” (1988a:142). Here is what Fromm said about Israel: “The claim of the Jews to the land of Israel cannot be a realistic political claim. If all nations would suddenly claim territories in which their forefathers lived two thousand years ago, this world would be a madhouse” (Woolfson 1980:13). As mentioned earlier, Marcuse, one of the principal leaders of the school, is on record advocating exactly the same policies for Israel as he advocated for majority-white-gentile countries. Marcuse suggested that Arabs who were displaced when Israel was created should return, even though “such a return would quickly transform the Jewish majority into a minority.” Marcuse explained:

[I]t is precisely the policy aiming at a permanent majority which is self-defeating. . . . To be sure, Israel would be able to sustain a Jewish majority by means of an aggressive immigration policy. . . . [L]asting protection for the Jewish people cannot be found in the creation of a self-enclosed, isolated, fear-stricken majority, but only in the coexistence of Jews and Arabs as citizens with equal rights and liberties (Marcuse 2005:181).

Again, by MacDonald’s own standards, this makes Marcuse *anti-Israel* and opposed to Jewish interests.

## Communism

One Jewish radical who is conspicuous for *not* being labeled an ethnic activist in *The Culture of Critique* is the most influential of them all: Karl Marx. Marx not only rejected his Jewish heritage, he went out of his way to express and promote viciously anti-Semitic views. In private correspondence, he smeared his socialist rival Ferdinand Lassalle (another Jew) with extremely anti-Semitic slurs and described Lassalle’s physical appearance, mannerisms, and habits as exemplifying unflattering Jewish characteristics (Gilman 1984:37). (The target of these obloquies, who was a major figure in his time, also held anti-Jewish views. In Lassalle’s words: “I do not like the Jews at all. I even detest them in general. . . . I have no contact with them”; Gilman 1984:37.) Most notorious is Marx’s 1844 essay, *On the Jewish Question*, which contains the famous lines: “What is the worldly religion of the Jew? *Huckstering.* What is his worldly God? *Money*. . . . An organization of society which would abolish the preconditions for huckstering, and therefore the possibility of huckstering, would make the Jew impossible” (Marx 2010:170).

According to MacDonald (1998a:54), Marx held that “Judaism, freed from the principle of greed, would continue to exist in the transformed society after the revolution (Katz 1986:113).” However, page 113 of Katz (1986) makes no reference or allusion of any kind to Marx or his ideas. In regard to Marx’s views on Jewish peoplehood, Katz (1986:122) cites only his view that (in Katz’s words) “Jews qua Jews would become liberated from their Judaism to take up their place as human beings in the socialist society of the future.”

Marx, Lassalle, and many other Jewish radicals who espoused anti-Semitic views might seem to be counterexamples to the thesis that Jews are uniquely ethnocentric. In any case, although MacDonald says little about Marx himself, he sees Marx-inspired ideologies, particularly Bolshevism, as bona fide Jewish intellectual movements designed to undermine gentile society and preserve Jewish separatism. The evidence for this claim, however, is not compelling.

MacDonald (1998a:80) cites Pipes’s (1993:112) suggestion that Jewish overrepresentation among Bolsheviks requires no special explanation because Jews were overrepresented in many fields—science, business, art, and so on. MacDonald rejects this idea with the following argument:

[E]ven assuming that these ethnically Jewish communists did not identify as Jews, such an argument fails to explain why such “de-ethnicized” Jews (as well as Jewish businessmen, artists, writers and scientists) should have typically been overrepresented in leftist movements and underrepresented in nationalist, populist, and other types of rightist political movements: Even if nationalist movements are anti-Semitic, as has often been the case, anti-Semitism should be irrelevant if these individuals are indeed completely deethnicized as Pipes proposes. Jewish prominence in occupations requiring high intelligence is no argument for understanding their very prominent role in communist and other leftist movements and their relative underrepresentation in nationalist movements.

This response to Pipes seems unconvincing. First, anti-Semitic nationalist movements generally targeted Jews regardless of their self-identity. Jews who identified as “Russian” or “Polish” would still have been discouraged, if not outright prohibited, from joining these movements as equal participants. Second, even “de-ethnicized” Jews might find it difficult to accept anti-Semitic caricatures of Jews due simply to their close contact with Jewish family and former friends.

For MacDonald, having a strong Jewish identity appears to be the only reason not to support anti-Semitic movements. As he says:

Even the most highly assimilated Jewish communists working in urban areas with non-Jews were upset by the Soviet-German nonaggression pact but were relieved when the German-Soviet war finally broke out . . .—a clear indication that Jewish personal identity remained quite close to the surface (1988a:62).

On the Nazi-Soviet nonaggression pact:

The nonaggression pact provoked a great deal of rationalization on the part of Jewish [Communist Party USA] members, often involving an attempt to interpret the Soviet Union’s actions as actually benefiting Jewish interests—clearly an indication that these individuals had not given up their Jewish identities. Others continued to be members but silently opposed the party’s line because of their Jewish loyalties (1988a:73).

Again, he interprets any objection to anti-Semitism—even silent opposition—as evidence that Jews are uniquely ethnocentric.

MacDonald devotes eight pages to “communism and Jewish identification in Poland” (1988a:61–69). A key claim in this section, based on work by Schatz (1991), is that the communist power structure was dominated by Jews seeking to preserve “Jewish group continuity in Poland while . . . destroy[ing] institutions . . . and . . . manifestations of Polish nationalism that promoted social cohesion among Poles” (1988a:68). He emphasizes repeatedly—based on Schatz (1991)—that the security service was devoted to this goal:

The core members of the security service came from the Jewish communists who had been communists before the establishment of the Polish communist government, but these were joined by other Jews sympathetic to the government and alienated from the wider society. . . . Jewish members of the internal security force often appear to have been motivated by personal rage and a desire for revenge related to their Jewish identity (MacDonald 1998a:66).

However, MacDonald leaves out a key fact noted by Schatz (1991:225), which is that 40% of the *victims* of the secret police were Jewish. Since the Jewish population of Poland at the time was miniscule (less than half of 1% of the population in 1949; see Schatz 1991:208), Jews were extremely disproportionately likely to be *attacked* by the security service. These data are more consistent with the thesis that Jews were simply more likely to be in positions of power—more likely to be in the position to persecute others, and more likely to be perceived as rivals by those in power, so more likely to be persecuted. There is no convincing evidence supporting the tale of Jews qua Jews victimizing gentiles for revenge on a significant scale.

## Diversity and Immigration

According to *The Culture of Critique*, “[the Jewish Horace] Kallen’s idea of cultural pluralism as a model for the United States was popularized among gentile intellectuals by John Dewey . . ., who in turn was promoted by Jewish intellectuals” (1988a:250). MacDonald points out that the editors of *Partisan Review* “published work by Dewey and called him ‘America’s leading philosopher’” and Dewey’s student, Sidney Hook, “was also unsparing in his praise of Dewey, terming him ‘the intellectual leader of the liberal community in the United States’” (1988a:250). Notice that, earlier, MacDonald argued that Margaret Mead was a puppet of her less-famous Jewish teacher, Boas. Here he argues that Dewey was being manipulated by his less famous, albeit Jewish, *student,* Sidney Hook. What is the reason why Dewey’s actions should be attributed to Jews?

Dewey was highly influential with the public at large. Henry Commager described Dewey as “the guide, the mentor, and the conscience of the American people; it is scarcely an exaggeration to say that for a generation no issue was clarified until Dewey had spoken” (in Sandel 1996:36). Dewey was the foremost advocate of “progressive education” and helped establish the New School for Social Research and the American Civil Liberties Union, both essentially Jewish organizations (MacDonald 1998a:250).

MacDonald concludes that Dewey “represented the public face of a movement dominated by Jewish intellectuals.”

Of course, any intellectual in late-nineteenth-to-mid-twentieth-century New York City was going to have a lot of Jewish associates. Where is the positive evidence that Dewey’s monumental success was the result of being propped up by Jewish ethnic activists? MacDonald (1988a:250) quotes Sandel’s (1996:35) opinion that Dewey’s “lack of presence as a writer, speaker, or personality makes his popular appeal something of a mystery.” But one explanation for Dewey’s success is that people were taken with his ideas rather than with his personal charisma. This would explain how he came to be extremely influential in China, where he was known as “a Second Confucius” (Grange 2004), even though there were no Jews there to promote him.

The President’s Commission on Immigration and Naturalization (1953:92–93) cited five experts who submitted testimony denying innate race differences in psychology. Two of these experts were Jewish (Ashley Montagu and Philip Hauser). One of the three gentiles was Mead. Another gentile—former president of the American Anthropological Association (AAA) Ralph L. Beals—reported that the AAA unanimously rejected innate race differences. To MacDonald (1998a:254), the followers of Boas were using the “ideology of racial equality [as] an important weapon on behalf of opening immigration up to all human groups.” (Boas himself died in 1942.)

An irony here is that the issue under consideration in this section of the commission’s report was not whether there were innate differences between whites and nonwhites, but whether there were innate differences between “Nordic” whites and *other whites* that justified limiting immigration from the latter group. The report cites Madison Grant:

The new immigration . . . contained a large and increasing number of the weak, the broken, and the mentally crippled of all [European] races drawn from the lowest stratum of the Mediterranean basin and the Balkans, together with hordes of the wretched, submerged populations of the Polish Ghettos. Our jails, insane asylums, and almshouses are filled with this human flotsam, and the whole tone of American life, social, moral, and political, has been lowered and vulgarized by them (President’s Commission on Immigration and Naturalization 1953:92).

MacDonald repeatedly cites Grant complaining that Jews opposed his (Grant’s) ideas. But in opposing the theory of Nordic superiority, Jews were effectively *promoting*, not undermining, white unity. Of course, many “Boasian” Jews argued that there were no differences between any races, but in the early twentieth century they were advocating immigration from all white countries whereas their opponents wanted to restrict immigration from non-Nordic white countries such as Italy and Poland.

According to *The Culture of Critique*, “American Jews have had no interest in proposing that immigration to Israel should be . . . multiethnic, or that Israel should have an immigration policy that would threaten the hegemony of Jews” (1988a:320). Regarding Jewish hypocrisy, MacDonald says:

Whereas American Jews have been in the forefront of efforts to ensure ethnic diversity in the United States and other Western societies, 40 percent of the [Israeli] Jewish respondents [in a 1988 survey reported in Smooha (1990:403)] agree that Israel should encourage Israeli Arabs to leave the country, 37 percent had reservations, and only 23 percent objected to such a policy. . . . Moreover, immigration to Israel is officially restricted to Jews (1988a:321).

Hypocrisy is when a person or group espouses values but applies them inconsistently. In order to attribute hypocrisy to people, it is necessary to identify the inconsistency *in those people*. MacDonald says that *American* Jews support multiculturalism, then cites a survey suggesting that many *Israeli* Jews advocate policies that are inconsistent with multicultural values. To justify the charge of hypocrisy it is necessary to find *individual* Jews advocating multiculturalism for the US and opposing it in Israel. Such Jews may exist, but there is no evidence that this is the norm. MacDonald treats the positions espoused by one Jew or group of Jews as a statement on behalf of the Jewish community and concludes that Jews are hypocrites because *different* Jews advocate policies that are mutually inconsistent. An alternative interpretation of the data is that Jews vary to some extent in their views among individuals and groups, in a way typical of other ethnicities.

Furthermore, the claim that immigration to Israel is restricted to Jews—even nominal Jews—was and is false. Since 1970, Israel will give automatic citizenship to anyone with one Jewish grandparent *and their non-Jewish spouse and children* (Israel Ministry of Foreign Affairs 2013). Hundreds of thousands of gentiles were granted Israeli citizenship because of this policy (Felter 2009). (An exact estimate is difficult to give since Israelis with no Jewish ancestors, or only a distant one, may identify as Jewish in surveys.)

It is also false that liberal Jews do not promote ethnic diversity in Israel. MacDonald says that Jews have supported black integration in the US “because such policies dilute Caucasian power and lessen the possibility of a cohesive, nationalist anti-Semitic Caucasian majority . . . while pursuing an anti-assimilationist, nationalist group strategy for their own group” (1988a:257). “American Jews have had no interest in proposing that immigration to Israel should be . . . multiethnic” (1988a:320). *The Culture of Critique* makes no mention of the fact that many of the same liberal Jews who advocate on behalf of blacks in the US pushed for the immigration of large numbers of nominally Jewish Ethiopians who have no genetic relation to other Jewish populations (Lucotte and Smets 1999). Israel, with around six million self-identified Jews (including at least a few hundred thousand who are not halachically Jewish), now contains a rapidly growing population of more than 135,000 Ethiopians (Myers-JDC-Brookdale Institute 2015). Alan Dershowitz, whom MacDonald (1998a:244, 318) identifies as an ethnic activist, played a leading role in pressuring the Israeli government to accept Africans who identify as Jews. In Dershowitz’s (2007) own words: “we have filed lawsuits, helped raise money, pressured leaders, and argued in the court of public opinion in favor of increased [multiethnic] immigration into Israel.”

At the end of *The Culture of Critique*, MacDonald asks what the ultimate consequences of Jewish-instituted liberal policies are likely to be in America. He suggests: “An important consequence—and one likely to have been an underlying motivating factor in the countercultural revolution—may well be to facilitate the continued genetic distinctiveness of the Jewish gene pool in the United States” (1988a:318). It is difficult to square this claim with the fact that Reform and unaffiliated Jews—the ones who participated in these liberal/multicultural movements—have an intermarriage rate of 50% and 69%, respectively (Pew Research Center 2013:37). (This may be an underestimate of intermarriage rates since Reform converts were counted as Jewish in Pew’s survey.) In fact, it is only those Jews who, as a group, were much less involved in national politics—the orthodox—who have low intermarriage rates and high fertility. MacDonald states that Jewish activists have increasingly started to see traditional Judaism as a better means of preserving group continuity. “Reform Judaism is becoming steadily more conservative, and there is a major effort within all segments of the Jewish community to prevent intermarriage. . . .” But contemporary Reform Judaism defines “intermarriage” as marriage between someone who *identifies* as a Jew and someone who *identifies* as a non-Jew. They care nothing for ethnic purity, and MacDonald never provides any evidence to the contrary.

## Conclusion: Evidence Favors the Default Hypothesis, and MacDonald Does Not Represent Evolutionary Psychology

MacDonald claims that several major twentieth-century liberal intellectual and political movements were consciously or unconsciously designed by Jews as part of a group evolutionary strategy to undermine gentile societies while preserving cohesion and continuity among themselves. High intelligence and ethnocentrism are supposedly genetic adaptations that help Jews pursue this strategy. According to the “default hypothesis” proposed in this paper, Jews, having relatively high mean levels of general intelligence and being concentrated in major cities, tend to be overrepresented in cognitively demanding fields, activities, and movements that are not overtly anti-Semitic, regardless of whether they are liberal or conservative. Anticipating this alternative explanation for Jewish overrepresentation in liberal movements, MacDonald seeks to protect his theory from being falsified by evidence that supports the default hypothesis:

As anti-Semitism develops, Jews begin to abandon the very movements for which they originally provided the intellectual impetus. This phenomenon may also occur in the case of multiculturalism. Indeed, many of the most prominent opponents of multiculturalism are Jewish neoconservatives, as well as organizations such as the National Association of Scholars (NAS), which have a large Jewish membership (1988a:313).

After arguing so strenuously that liberal movements were designed to advance a Jewish group evolutionary strategy, he acknowledges that Jews are also in the vanguard in the fight against those same movements.

In recent years, Jews have continued to produce examples favoring the default hypothesis. The most high-profile opponent of liberal activism in social science is, *without question,* Jonathan Haidt (see Duarte et al. 2015), who is Jewish. The most high-profile advocate of incorporating Darwinism into the social sciences is another Jew, Steven Pinker (e.g., Pinker 2002). The Foundation for Individual Rights in Education (FIRE)—the most prominent organization that defends free speech on campus, primarily the speech of conservatives—was founded by Alan Charles Kors and Harvey A. Silverglate, both Jewish. There is reason to believe that Jews played a significant role in Donald Trump’s election and, specifically, in his anti-immigration policies (Dolsten 2017). The term “paleoconservative,” referring to a pro-white, pro-Western-tradition political doctrine, was coined by Herbert Marcuse’s PhD student Paul Gottfried, who is Jewish. Gottfried was also the first person to publicly use the term “alternative right” to refer to a race-conscious conservatism that opposes immigration and multiculturalism (Siegel 2016). The only major white nationalist organization that is not anti-Semitic is American Renaissance. Out of the 10 invited speakers at the first American Renaissance conference in 1994, four were Jewish (American Renaissance 2017).

Salter (2000) notes that many of the sources cited in *The Culture of Critique* are “mainstream.” Indeed, while the Judaism-as-a-group-evolutionary-strategy trilogy has the accoutrements of sound scholarship, such as detailed endnotes and extensive bibliographies to sources that are *themselves* credible, the evidence reviewed here suggests that this is a smokescreen. MacDonald’s theory is built on misrepresented sources, cherry-picked facts, and assiduous refusal to consider the more parsimonious default hypothesis that the evidence actually supports.

Finally, let us turn to the question of MacDonald’s relationship with evolutionary psychology. Some opponents of evolutionary psychology have taken his conclusions as the more or less inevitable outcome of applying evolutionary theory to Judaism. Some *supporters* of evolutionary psychology (such as D. S. Wilson and others listed earlier) have also claimed that MacDonald is correctly applying evolutionary psychological thinking to Judaism. But misrepresenting sources and distorting history are not part of the methods of evolutionary psychology, or any other legitimate academic discipline. Those who have competently applied insights from evolutionary theory to the study of Judaism (e.g., Goldberg 2009; Konner 2003) have come to very different conclusions than we find in *The Culture of Critique*. It is they, not MacDonald, who should be treated as representatives of evolutionary psychology and biosocial science.

## Footnotes

1 MacDonald’s (1988a:154, n. 15) list of the four who were not influenced by Freud does not include Silvers.
